# Analysis of p16 gene mutations and deletions in childhood acute lymphoblastic leukemias

**DOI:** 10.1590/S1516-31802003000200005

**Published:** 2003-03-05

**Authors:** José Alexandre Rodrigues Lemos, Ricardo Defavery, Carlos Alberto Scrideli, Luiz Gonzaga Tone

**Keywords:** Acute, Lymphoblastic, Leukemia, Tumor, Suppressor, Gene, p16, Leucemia, Linfoblástica, Aguda, Gene, Supressor, Tumor, p16

## Abstract

**CONTEXT:**

The p16 tumor suppressor gene encodes a cyclin-dependent kinase 4 inhibitor that blocks cell division during the G1 phase of the cell cycle. Alterations in this gene have been reported for various neoplasia types, including acute lymphoblastic leukemias (ALL), especially T-cell acute lymphoblastic leukemias (ALL).

**OBJECTIVE:**

To determine probable alterations in the p16 gene in children with acute lymphoblastic leukemias using the polymerase chain reaction (PCR) and direct DNA sequencing and also to analyze event-free survival (EFS).

**DESIGN:**

Retrospective study.

**SETTING:**

Department of Child Care and Pediatrics, Faculty of Medicine of Ribeirão Preto, Universidade Federal de São Paulo.

**PARTICIPANTS:**

Fifty-six children with ALL (mean age 4 years). Forty (71.43%) had B-cell and 12 (21.43%) had T-cell ALL; 4 (7.1%) were biphenotypic.

**SAMPLE:**

DNA samples were extracted from bone marrow upon diagnosis and/or relapse. In 2 T-cell cases, DNA from cerebrospinal fluid (CSF) was analyzed.

**MAIN MEASUREMENTS:**

Deletions or nucleotide substitutions in exons 1, 2 and 3 of the p16 gene were determined by PCR and nucleotide sequencing. Event-free survival was determined by the Kaplan-Meyer and log-rank test for patients carrying normal and altered p16.

**RESULTS:**

Deletions in exon 3 were observed in five cases. Abnormal migration in PCR was observed in seven cases for exon 1, six for exon 2, and five for exon 3. Mutations in exon 1 were confirmed by direct DNA sequencing in four cases and in exon 2 in two cases. The Kaplan-Meyer survival curves and the log-rank test showed no significant differences in 5-year EFS between children with normal or altered p16, or between patients with BALL carrying normal or altered p16 gene. Patients with T-ALL could not be evaluated via Kaplan-Meier due to the small number of cases.

**CONCLUSIONS:**

Our results, particularly regarding deletion frequency, agree with others suggesting that deletions in the p16 are initial events in leukemia genesis. The small number of samples did not allow stablishment of correlation between childhood ALL and the p16 point mutations found in our study. Kaplan-Meier analysis revealed no significant correlation between EFS and alterations in ALL. The p16 alterations frequency observed for B and TALL agreed with reports from other centers.

## RESUMO

**CONTEXTO:**

O gene supressor tumoral p16 codifica uma proteína inibidora da quinase ciclina dependente 4(cdk4) que bloqueia a divisão celular na fase G1 do ciclo celular. Alterações neste gene foram relatadas em várias neoplasias, incluindo as leucemias linfoblásticas agudas, principalmente da linhagem T.

**OBJETIVO:**

Verificar prováveis alterações do gene p16 em leucemias linfoblásticas agudas em crianças, utilizando o método de reação de polimerase em cadeia - polimorfismo conformacional em fita simples e seqüenciamento direto de nucleotídeos e comparar a sobrevida livre de eventos com a presença ou não de alterações (deleções ou substituições de bases) do gene p16, utilizando o método de Kaplan-Meir e teste log-rank.

**TIPO DE ESTUDO:**

Estudo retrospectivo.

**LOCAL:**

Departamento de Puericultura e Pediatria da Faculdade de Medicina de Ribeirão Preto da Universidade de São Paulo.

**PARTICIPANTES:**

Foram estudadas 56 crianças (16 do sexo feminino e 40 do masculino) com leucemia aguda com idade média de quatro anos. Quarenta pacientes (71,43%) tinham leucemias linfoblásticas agudas de células B, 12 (21,43%) tinham leucemias linfoblásticas agudas de células T e 4 (7,1%) eram bifenotípicas.

**AMOSTRA:**

Amostras de DNA foram extraídas de medula óssea obtidas ao diagnóstico e/ou na recaída. Em dois casos de leucemias linfoblásticas agudas de células T, o DNA obtido do líquido céfalo-raquidiano foi analisado.

**VARIÁVEIS ESTUDADAS:**

Deleções ou subs-tituições de bases nos éxones 1, 2 e 3 do gene p16 foram determinadas pela reação de polimerase em cadeia - polimorfismo conformacional em fita simples e seqüenciamento de nucleotídeos. A sobrevida livre de eventos foi determinada segundo o método de Kaplan-Meier e teste log-rank para pacientes com gene p16 normal e alterado.

**RESULTADOS:**

Em cinco casos (quatro leucemias linfoblásticas agudas pré B CALLA+ e uma leucemia linfoblástica aguda T) foram observadas deleções no éxon 3. Foram verificadas migrações anômalas no polimorfismo conformacional em fita simples em sete casos no éxon 1, seis no éxon 2 e cinco no éxon 3. A confirmação da mutação pelo seqüenciamento direto do DNA ocorreu em quatro casos no éxon 1 e 2 no éxon 2. Foram comparadas as curvas de sobrevida pelo método de Kaplan-Meier e teste log-rank e não se obteve diferença estatisticamente significativa da sobrevida livre de eventos em cinco anos entre os grupos de criança com leucemia linfoblática aguda com p16 normal e alterado e também entre os pacientes com leucemia linfoblástica aguda B com p16 normal e alterado. Não foi possível avaliar pelo método de Kaplan-Meier os pacientes com leucemia linfoblástica aguda T devido ao pequeno número de casos.

**CONCLUSÕES:**

Os resultados obtidos, principalmente em relação à freqüência de deleções, foram concordantes com vários autores que sugerem que deleções do gene p16 possam ser eventos iniciais da gênese leucêmica. Tendo em vista o pequeno número de amostras estudadas, não foi possível realizar a correlação entre leucemia linfoblática aguda na infância e as mutações de ponto no gene p16 encontradas neste estudo. Não se obteve significância estatística entre sobrevida livre de eventos e alterações do p16 em leucemia linfoblática aguda pelo método de Kaplan-Meier. A freqüência de alterações do p16 em leucemia linfoblástica aguda B e T foram concordantes com os estudos realizados em al-guns centros mundiais. Sugerimos a realização de uma pesquisa multicêntrica com um maior número de casos, principalmente sobre leucemia linfoblástica aguda T, a fim que seja possível traçar um perfil de alterações do gene p16 em crianças brasileiras.

## INTRODUCTION

The cell cycle, a complex process in which a large number of regulatory proteins is involved, can suffer alterations caused by different factors that can consequently transform a normal cell into a malignant one. These alterations are the result of the gradual accumulation of different genetic events, the main ones being the activation of oncogenes and the inactivation of tumor suppressor genes. These events, in turn, can lead to uncontrolled cell proliferation and the occurrence of neoplasia.^1,3^

The p16 tumor suppressor gene located on the short arm of chromosome 9 encodes a cyclin-dependent kinase 4 (cdk4) inhibitor that blocks cell division during the G1/S phase of the cell cycle by forming a complex that promotes the dissociation of cyclin from the kinase. The cyclin-dependent kinase 4 and cyclin-dependent kinase 6/cyclin D complexes play an important role in the phosphorylation of the RB protein. Therefore, an increase in p16 expression in cells with a normal RB gene results in the blockade of the G1 phase, indicating the inhibitory function of the p16 gene in the cell cycle as well as its role in neo-plastic processes.^4,5^

Recently, greater importance has been attributed to the role of p16 gene alterations in human neoplasia, mainly as a result of the high frequency of such alterations observed for cell lines derived from different tumors and of the direct involvement of this gene in cell cycle regulation.^4-8^

In acute lymphoblastic leukemia (ALL), inactivation of the p15 and p16 genes represents one of the most common genetic mechanisms of tumorigenesis.^9^ Some authors have suggested that alterations in the p16 gene are associated with a higher frequency of relapse and lower survival in T-cell acute lymphoblastic leukemias both in adults^10^ and children.^11-13^ Therefore, p16 inactivation might be of prognostic value in acute lymphoblastic leukemias, especially T-cell acute lymphoblastic leukemias, playing an important role in its genesis or evolution.^6,11,14-24^

The most frequent alterations in the p16 gene are total or partial homozygous deletions. These alterations are more frequently observed for T-cell acute lymphoblastic leukemias and are less likely in B-cell acute lymphoblastic leukemias. However, the reported frequency of these mutations is highly variable, ranging from 0 to 100% depending on the origin of the patient.^12,17,18,23-31^ In acute lymphoblastic leukemias, the frequency of nucleotide substitutions in the p16 gene is estimated to be approximately 8%, ranging from 0 to 5% for B-cell acute lymphoblastic leukemias and from 0 to 13% for T-cell acute lymphoblastic leukemias.^12,16,18,19,25,28,29^

Based on the importance of the p16 gene in acute lymphoblastic leukemias, especially T-cell acute lymphoblastic leukemias, the aim of the present study was to determine probable alterations in the p16 gene in Brazilian children with acute lymphoblastic leukemias using the polymerase chain reaction-single stranded conformation polymorphism (PCRSSCP) method and direct DNA sequencing and also to compare the event-free survival (EFS) using the Kaplan-Meier method and the log-rank test for patients carrying normal or altered p16 genes.

## METHODS

DNA was extracted from the bone marrow of 56 children with acute lymphoblastic leukemias treated at the Pediatric Oncology and Hematology Service, Hospital Universitário, Faculdade de Medicina de Ribeirão Preto, Universidade de São Paulo (HCFMRPUSP), during the period from 1991 to 1997.

Sixteen (28.57%) of the 56 children studied were females and 40 (71.43%) were males. Immunophenotyping of the patients showed the following distribution: 40 children had B-cell acute lymphoblastic leukemias (71.43%), 12 had T-cell acute lymphoblastic leukemias (21.43%), and 4 had biphenotypic acute lymphoblastic leukemias (7.14%). Sixty-two samples of DNA from bone marrow collected at diagnosis and/or relapse and two samples from cerebrospinal fluid (CSF) from two central nervous system cases collected at relapse were analyzed via the polymerase chain reaction. DNA samples were obtained from 40 patients (25 B-acute lymphoblastic leukemias, 11 T-acute lymphoblastic leukemias and 4 biphenotypic) at diagnosis only, from 8 patients (B-acute lymphoblastic leukemias) at diagnosis and relapse, and from 8 patients at relapse only (seven B-acute lymphoblastic leukemias and one T-acute lymphoblastic leukemia). Patient ages at diagnosis ranged from 6 months to 14 years, with a mean age of 4 years. Acute lymphoblastic leukemia was classified according to immunophenotype criteria via cytofluorometric analysis using monoclonal antibodies carried out at the Hematology Laboratory.

To verify the presence of probable deletions or base substitutions in the p16 gene, the samples of DNA obtained from children with acute lymphoblastic leukemias at diagnosis and/or relapse were tested by the polymerase chain reaction-single stranded conformational polymorphism method. A control amplification was carried out using the β-globin gene (600-bp fragment) via multiplex polymerase chain reaction for those polymerase chain reaction products that showed no amplification of the exon, in order to confirm deletion The samples showing abnormal migration upon single-stranded conformational polymorphism were submitted to nucleotide sequencing to confirm the probable mutation.

The polymerase chain reaction products of the bone marrow samples were analyzed by single-stranded conformational polymorphism for possible point mutations in exons 1, 2 and 3 of the p16 gene using corresponding primer pairs (Table 1).^17,30^ For the polymerase chain reaction, each DNA sample (0.1 μg/μl) was added to a mixture containing 2.5 mM buffer (0.2 M Tris-HCl, 0.5 M KCl, pH 8.4), 10 mM dNTPs (dATP, dCTP, dGTP, dTTP), 50 mM MgCl_2_, 1% DMSO, 10 μg/μl of the primer pair corresponding to the exon studied, and 5 U/μl Taq polymerase, in a final volume of 25 μl. All samples were submitted to the following amplification conditions (for all exons): 35 successive denaturation cycles (1 minute at 94° C), annealing at 58° C for 1 minute, and extension at 72° C for 1 minute.

**Table 1:** Primers used, and characteristics and regions of the p16 gene analyzed by single-stranded comformational polymorphism in bone marrow samples

Primer	Sequence	Sense/Antisense	Exon	Exon (bp)	Sequence (bp)
P1611	5'CGGAGAGGAGAG3’	Sense	1	126	276
P1612	5'GCGCTACCTGATTCCAATTC3'	Antisense	1		
P1621	5'TTCCTTTCCGTCATGCCG3’	Sense	2	307	394
P1622	5'GTACAAATTCTCAGATCATCAGTCCTC3'	Antisense	2		
P1631	5'GTTTTCTTTCTGCCCTCTGC3’	Sense	3	14	347
P1632	5'CCCACATGAATGTGCGCTT3’	Antisense			

For single-stranded conformational polymorphism, the polymerase chain reaction products were diluted 1:10 in a solution containing 0.1% SDS (Sodium Dodecyl Sulfate) and 10 mM EDTA (Ethylene diamine tetraacetic acid) and an equal volume of dye solution containing 20 mM EDTA, 95% formamide, 0.05% bromophenol blue and 0.05% xylene cyanol were added. The samples were denatured at 96° C for 10 minutes and a fraction was applied to non-denaturing 6% polyacrylamide gel and submitted to electrophoresis in 1X TBE buffer for 5 hours at 8 watts. The gel was stained with silver nitrate, developed, photographed and analyzed.

A 100-μl aliquot of each polymerase chain reaction product was used for DNA purification for direct sequencing of the samples that showed an abnormal electrophoretic migration pattern upon polymerase chain reaction-single stranded conformational polymorphism. After DNA purification, samples were submitted to direct sequencing using the T7 kit (Pharmacia) and 6% non-denaturing polyacrylamide gel electrophoresis. The gel was then dried on filter paper and exposed to an X-ray film. The sequences obtained for each sample were compared to the normal SEG–HSPCDK[2182138] sequence consisting of U12818 (exon 1), U12820 (exon 2) and U12820 (exon 3).

Event-free survival was determined for patients carrying the normal or altered p16 gene by the Kaplan-Meier method. Curves were compared by the log-rank test. Statistical analysis was carried out using the GraphPad Prism software, version 2.0 (GraphPad Software Inc., San Diego, CA).

The study was approved by the Ethics Committee of HCFMRP-USP (case no. 5800/2001).

## RESULTS

Sixty-two bone marrow DNA samples obtained at diagnosis and/or relapse and two cerebrospinal fluid samples obtained at relapse from 56 children with acute lymphoblastic leukemias were analyzed. In six DNA samples from five patients that were not amplified by polymerase chain reaction when tested with exon 3 primers, deletions were confirmed by multiplex reaction simultaneously with the ß-globin primers. The deletions detected were in four pre-B CALLA+ acute lymphoblastic leukemias and one T-acute lymphoblastic leukemia (Table 2). In three cases (two pre-B CALLA+ acute lymphoblastic leukemias and one T-acute lymphoblastic leukemia), the deletions were observed only at diagnosis, in one they were observed at diagnosis and relapse, and in the last one only at the second relapse.

**Table 2:** Base substitutions compared to the SEG_HSPCDK [2182138] sequence

Patient	Immunophenotype	Mutation	Position		Codon		Amino acid		Phase	Material	Exon
			Base	AA	Wild type	Mutated	Wild type	Mutated			
L147	Pre-pre-B	C > T	367	123	CGC	TGC	Arg	Cys	Diagnosis	BM	2
L023	CALLA+	A > C	107	36	TAC	TCC	Tyr	Ser	2nd relapse	BM	1
L126	CALLA+	A > C	101	34	AAT	ACT	Asn	Thr	Diagnosis	BM	1
L325	CALLA+	C > T	95	32	GCA	GTA	Ala	Val	Diagnosis	BM	1
L339	CALLA+	G > T	94	32	GTA	TCA	Ala	Ser	Diagnosis	BM	1
L341	T	T > A	351	117	GAT	GAA	Asp	Glu	CNS relapse	CSF	2

Abnormal migration in the single-stranded conformational polymorphism method was observed in seven cases for exon 1, in six for exon 2 and in five for exon 3.

All samples showing abnormal behavior upon single-stranded conformational polymorphism were submitted to direct DNA sequencing. Base substitution in exon 1 was confirmed in four cases; two cases in exon 2 and none in exon 3 (Table 2).

Forty-eight patients with acute lymphoblastic leukemias were analyzed regarding the event-free survival using Kaplan-Meier curves and the log-rank test, by comparing a group of children with a normal p16 gene (n = 41) with a group of children carrying the altered p16 gene (deletion or mutation) (n = 7). The 5-year event-free survival did not differ between the two groups (p = 0.548). In addition, no difference in the 5-year event-free survival between the two groups was observed for children with B-cell acute lymphoblastic leukemias (p = 0.73). Event-free survival could not be determined separately for the group with T-cell acute lymphoblastic leukemias, due to the small size of the sample.

## DISCUSSION

Great advances have been made over the last few years in the understanding and treatment of childhood cancer,^32^ especially in studies determining possible genetic alterations in tumor suppressor genes and oncogenes. These have allowed more precise diagnoses and the determination of important tumor markers for the evolutive analysis of neoplasia.

The p16 gene was first described by Serrano et al.^5^ in 1993, and since then numerous studies on this gene have been published. In our work, 56 children with acute lymphoblastic leukemias were studied by polymerase chain reaction-single stranded conformational polymorphism and direct DNA sequencing at diagnosis and/or relapse in order to determine possible alterations in the p16 gene and their biological role in this disease.

Deletions were only observed in the exon 3 region of the DNA samples analyzed, and were identified in 10% (4/40) of B-cell acute lymphoblastic leukemia cases and 8% (1/12) of T-cell acute lymphoblastic leukemia cases (Table 2). According to several authors, deletions are more frequent in T-cell acute lymphoblastic leukemias than in B-cell acute lymphoblastic leukemias.^12,17,18,,20,21,24-31^ However, the frequency of p16 gene deletions in acute lymphoblastic leukemias is generally heterogeneous, especially in T-cell acute lymphoblastic leukemias, ranging from 0%^33^ to 100% for the latter,^24^ and from 4%^34^ to 50% for B-cell acute lymphoblastic leukemias.^24^ This heterogeneity seems to be related to the origin of the patient, since frequencies vary according to the place where the studies are carried out. The results obtained in the present study for B-cell acute lymphoblastic leukemias are similar to those found in Australia (Perth),^35^ Germany,^34^ and France (Paris).^25^ The 8% incidence of deletions in T-cell acute lymphoblastic leukemias observed in the present study can be considered to be low and is compatible with frequencies obtained in studies carried out in Chicago (0%)^32^ and Japan (15%).^29^ According to Cayuela et al.,^25^ the reason for the variation in p16 gene deletions has yet to be established. Since Ribeirão Preto, Brazil, has been undergoing intense migration and has a population of many different ethnic origins, we cannot conclude that our data are representative of the Ribeirão Preto region, especially considering that variables such as race and family origin were not analyzed.

Of the 18 cases for whom an abnormal migration pattern could be demonstrated upon single-stranded conformational polymorphism, nucleotide substitutions were confirmed in only six cases by direct nucleotide sequencing (four of exon 1 and two of exon 2). The occurrence of false abnormal bands might be due to three factors. First, electrophoresis carried out at low temperatures produces better quality bands but with a greater probability of false bands compared to electrophoresis carried out at room temperature.^36^ Second, it is possible that mutations occurred in the polymorphic regions of non-coding introns adjacent to the exon. Finally, errors in single-stranded conformational polymorphism analysis cannot be excluded, since bands that might be interpreted as subtle alterations are indeed normal bands.

Of the 56 patients studied, 11% presented nucleotide substitutions, with 5 out of 40 patients (12%) having B-cell acute lymphoblastic leukemias and 1 out of 12 (8%) having T-cell acute lymphoblastic leukemias. Of the 6 cases presenting nucleotide substitutions, 4 were detected at diagnosis and 2 at relapse. Three patients (one early pre-B acute lymphoblastic leukemias and two pre-B CALLA + acute lymphoblastic leukemias) died and three (two pre-B CALLA+ acute lymphoblastic leukemias and one T-cell acute lymphoblastic leukemia) are currently in remission. According to Dicciani et al.,^12^ it can be supposed that the mutations detected at diagnosis in the present study are probably related to early events in acute lymphoblastic leukemias. The following previously undescribed mutations were observed in four of our patients with nucleotide substitutions: GCA^Ala^ > GTA^Val^ and GTA^Ala^ > TCA^Ser^, both in codon 32 (cases L325 and L339), AAT^Asn^ > ACT^Thr^ in codon 34 (case L126), and GCC^Agr^ > TGC^Cys^ in codon 123 (case L147). Since data on mutations in childhood acute lymphoblastic leukemias are scarce, it is difficult to establish whether these mutations play a role in the inactivation of the p16 gene or are polymorphisms. In the present study, TAC^Tyr^ > TCC^Ser^ substitution was observed in codon 36 in a patient with pre-B CALLA+ acute lymphoblastic leukemias. In the literature, mutations in codon 36 have been described in 2 cases of adult lung cancer.^37,38^ In another case from our study (T-cell), a mutation was observed in a patient (L341) with central nervous system relapse whose bone marrow was normal and for whom DNA was isolated from the cerebrospinal fluid. Yoshida et al.^39^ observed nucleotide substitutions in the same codon, which produced a silent mutation in biliary tract carcinoma. Authors such as Otsuki et al.^18^ and Nakao et al.^28^ found low frequencies of nucleotide substitutions in the p16 gene in T-cell acute lymphoblastic leukemias, while others^17,26^ did not observe any mutation in T-cell acute lymphoblastic leukemias.

In the present study, analysis of all immunophenotypes as a whole did not reveal any difference in the event-free survival of children with acute lymphoblastic leukemias who carried a normal or altered p16 gene, similar to the findings of Rubnitz et al.^31^ With respect to children with B-cell acute lymphoblastic leukemias, there was also no difference in event-free survival between the two groups (p = 0.73), in agreement with other studies.^11,12,33,40^ We were unable to analyze T-cell acute lymphoblastic leukemias due to the small number of patients with this immunophenotype. However, 2 patients with alterations in the p16 gene showed early relapse.

According to several authors, p16 inactivation may be an important factor for the assessment of the recurrence risk, especially in children with T-cell acute lymphoblastic leukemias.^11-14^ In the present study, no correlation could be established between the nucleotide substitutions found in six patients and patient survival or prognosis, probably due to the low frequency of mutations in this gene in childhood leukemias.

## CONCLUSION

In conclusion, despite the small number of patients, especially T-cell acute lymphoblastic leukemia cases, the present study provides a first analysis of the p16 gene in Brazilian patients with childhood leukemia. For consistent conclusions, a Brazilian multicenter study including a larger number of cases, especially T-cell acute lymphoblastic leukemia patients, would be fundamental in order to compare possible alterations in the p16 gene with the origin of Brazilian patients with acute lymphoblastic leukemias.
